# Early application of continuous high-volume haemofiltration can reduce sepsis and improve the prognosis of patients with severe burns

**DOI:** 10.1186/s13054-018-2095-9

**Published:** 2018-07-06

**Authors:** Bo You, Yu Long Zhang, Gao Xing Luo, Yong Ming Dang, Bei Jiang, Guang Tao Huang, Xin Zhu Liu, Zi Chen Yang, Yu Chen, Jing Chen, Zhi Qiang Yuan, Su Peng Yin, Yi Zhi Peng

**Affiliations:** 1 State Key Laboratory of Trauma, Burns and Combined Injury, Institute of Burn Research, Southwest Hospital, Third Military Medical University (Army Medical University), Chongqing, China; 2 Department of Cardiothoracic Surgery, No. 324 Hospital of PLA, Chongqing, China; 3 Department of Plastic Surgery, No. 474 Hospital of PLA, Urumqi, China

**Keywords:** Severe burn, Blood purification, High-volume haemofiltration, Inflammatory cytokine, Immunocyte, Sepsis

## Abstract

**Background:**

In the early stage of severe burn, patients often exhibit a high level of inflammatory mediators in blood and are likely to develop sepsis. High-volume haemofiltration (HVHF) can eliminate these inflammatory mediators. We hypothesised that early application of HVHF may be beneficial in reducing sepsis and improving the prognosis of patients with severe burns.

**Methods:**

Adults patients with burns ≥ 50% total burn surface area (TBSA) and in whom the sum of deep partial and full-thickness burn areas was ≥ 30% were enrolled in this randomised prospective study, and they were divided into control (41 cases) and HVHF (41 cases) groups. Patients in the control group received standard management for major burns, whereas the HVHF group additionally received HVHF treatment (65 ml/kg/h for 3 consecutive days) within 3 days after burn. The incidence of sepsis and mortality, some laboratory data, levels of inflammatory cytokines in the blood, HLA-DR expression on CD14^+^ peripheral blood monocytes, the proportion of CD25^+^Foxp3^+^ in CD4^+^ T lymphocytes, and the counts of CD3^+^, CD4^+^ and CD8^+^ T lymphocytes were recorded within 28 days post-burn.

**Results:**

The incidence of sepsis, septic shock and duration of vasopressor treatment were decreased significantly in the HVHF group. In addition, in the subgroup of patients with burns ≥ 80% TBSA, the 90-day mortality showed significant decreases in the HVHF group. The ratio of arterial oxygen partial pressure to the fraction of inspiration oxygen was improved after HVHF treatment. In the patients who received HVHF treatment, the blood levels of inflammatory cytokines, including tumour necrosis factor-α, interleukin (IL)-1β, IL-6 and IL-8, as well as the blood level of procalcitonin were found to be lower than in the control group. Moreover, higher HLA-DR expression on CD14^+^ monocytes and a lower proportion of CD25^+^Foxp3^+^ in CD4^+^ T lymphocytes were observed in the patients in the HVHF group.

**Conclusions:**

Early application of HVHF benefits patients with severe burns, especially for those with a greater burn area (≥ 80% TBSA), decreasing the incidence of sepsis and mortality. This effect may be attributed to its early clearance of inflammatory mediators and the recovery of the patient’s immune status.

**Trial registration:**

Chinese Clinical Trial Register, ChiCTR-TRC-12002616. Registered on 24 October 2012.

**Electronic supplementary material:**

The online version of this article (10.1186/s13054-018-2095-9) contains supplementary material, which is available to authorized users.

## Background

Sepsis is an unsolved medical problem worldwide. Sepsis and septic shock occur in millions of people around the world each year, and one in four (and often more) of them die [1]. Sepsis is a leading cause of death among patients with severe burns, particularly when it is complicated by septic shock or multiple organ dysfunction syndrome (MODS). Once septic shock or MODS occurs, no specific and effective therapeutic measures are available, resulting in a very poor prognosis. Patients with severe burns often exhibit an intense stress response and produce large amounts of inflammatory mediators, which are prone to result in organ damage and immune dysfunction, increasing susceptibility to infections and even causing sepsis [2, 3]. Theoretically, the early blockade or inhibition of excessive inflammatory reactions may improve a patient’s condition. However, because the cytokine network is unlikely to control the systemic inflammatory response by the simple blockade or elimination of some specific meditators, previous clinical studies on the antagonisation of some inflammatory mediators did not demonstrate an associated benefit to patients [4–6]. Therefore, effective therapeutic measures for sepsis remain lacking.

Blood purification can non-specifically eliminate inflammatory mediators in blood, controlling the levels of pro-inflammatory and anti-inflammatory mediators and regulating the host immune responses by means of filtration, adsorption and plasma exchange [7]. Therefore, blood purification has been applied as an organ support therapy for critically ill patients, including those with sepsis [7]. Currently, advances in blood purification render it a promising therapeutic measure for treating sepsis [8–10].

However, it remains controversial whether blood purification can improve the prognosis of patients with sepsis [1, 11]. Our previous work has verified that continuous venovenous haemofiltration (CVVH) can improve organ function, maintain homeostasis and relieve inflammatory reactions in burn patients with sepsis but cannot improve their survival rate [12, 13]. In clinical work, we also found that the effect of using blood purification as a salvage therapy on burn patients with septic shock and MODS was not as good as expected. Microcirculatory disorders and organ functional failure in patients with septic shock, who often exhibit refractory shock, severe metabolic acidosis, hypoxemia and increased lactic acid, are difficult to reverse using blood purification. A recent multi-centre randomised controlled trial showed that HVHF was effective in reversing shock and improving organ function over a 2-week period in burn patients with septic shock and acute kidney injury (AKI), but that it was ineffective in decreasing cytokine levels and improving survival [14]. Possible reasons for this may be that the excessive inflammatory reactions that occur during sepsis or septic shock were far beyond the clearance capacity of current blood purification methods or that the patients had already experienced irreversible secondary organ injury and microcirculatory and mitochondrial dysfunction [14]. These blood purification interventions might be too late following the onset of sepsis or septic shock.

Therefore, patients may benefit from blood purification when applied at the early stage of burns rather than when MODS and sepsis occur. Theoretically, blood purification is effective during the early stage of severe burns owing to its ability to non-specifically remove a broad spectrum of inflammatory mediators, stress hormones, oxygen radicals, myoglobin, metabolites and toxins; to regulate water-electrolyte and acid-base equilibria; to clear excessive water in vivo (especially the clearance of third space liquid); and to relieve tissue oedema and improve the microcirculation in organs [15]. Studies have shown that in patients with burns with a total burn surface area (TBSA) > 40% complicated by AKI, the early application of CVVH can reduce mortality and improve the clinical symptoms of patients in cases that are complicated by shock and acute lung injury/acute respiratory distress syndrome, which may be associated with an early reduction of inflammatory mediators [16]. Therefore, we hypothesised that the early application of continuous blood purification to patients with severe burns might regulate immune function, reduce the incidence of sepsis and improve patient prognosis through the clearance of excessive inflammatory mediators.

High-volume haemofiltration (HVHF), which has evolved from renal replacement therapy (RRT), enhances the convection and adsorption on medium-molecular-weight solutes, and improves the clearance ability by increasing the input of the displacement liquid. Therefore, it is regarded as a common and effective blood purification technique for the treatment of inflammatory mediator-related diseases and may improve outcomes in systemic inflammatory response syndrome and sepsis [11, 17]. In the present study, we explored the early application of HVHF therapy in patients with burns in a randomised prospective study, and observed the levels of several inflammatory cytokines in the blood circulation, immune phenotypes in peripheral blood immune cells, and the clinical outcomes.

## Methods

### Patients

This randomised trial was approved by the ethics committee of Southwest Hospital, Third Military Medical University (Army Medical University), and was performed between March 2014 and October 2017. The inclusion criteria were as follows: (1) aged 18 to 65 years, (2) burns ≥ 50% TBSA, and (3) the total surface area of deep burns (deep partial thickness and full-thickness burns) ≥ 30%. Patients were excluded for any of the following reasons: (1) admission more than 3 days after burn; (2) patients with sepsis or multiple organ failure (MOF), which was defined as organ failure (score ≥ 3 points) of at least two of the organs or systems according to the Sequential Organ Failure Assessment (SOFA) score [18]; (3) documented past history of chronic organ system insufficiency, defined as history of heart failure, cirrhosis, chronic lung disease and receiving chronic dialysis, according to evaluation by the Acute Physiology and Chronic Health Evaluation II (APACHE II) score [19]; (4) pregnant women or lactating patients; (5) patients with mental diseases or immune functional defects; and/or (6) patients with poor compliance for whom completion of treatment was found to be difficult. Withdrawal criteria included the following: patients who abandoned therapy and were discharged against medical advice during the observation period. Patients who met the inclusion criteria and voluntarily participated in the trial or whose legal representatives signed informed consent forms were then randomised into two groups according to a randomised digit table.

### Control group

After admission, all patients received standard care for burns [20, 21], which included the following:

1. First aid
2. Fluid resuscitation, resuscitation with crystalloids followed by colloids according to the Third Military Medical University protocol [22] (target hourly urine output greater than 0.5 ml/kg/h), as well as haemodynamic monitoring by pulse-indicated continuous cardiac output when necessary
3. Management of inhalation injury
4. Early excision and skin graft, which were performed after fluid resuscitation at 3–7 days post-burn [23]; the covering includes autologous skin grafts, meshed grafts, MEEK grafts, micro-skin autografts overlaid with large sheet allograft, single large sheet allograft, or heterogenetic porcine skin grafts
5. Glucose control
6. Prevention and treatment of infection by systemic administration of antibiotics
7. Prevention of stress ulcer
8. Early enteral nutrition
9. Organ support therapy
10. Bedside rehabilitation training

### Intervention

In addition to the therapies given to the patients in the control group as described above, vascular access was obtained using a double-lumen dialysis catheter inserted into the femoral vein by Seldinger’s technique, and HVHF therapy was performed within 3 days after the burn using the PRISMAFLEX System (Gambro Lundia AB, Lund, Sweden) with a 1.5-m^2^ haemofilter (AN69, M150 Set; Gambro Industries, Meyzieu Cedex, France). Blood flow was set between 200 and 250 ml/min, and the ultrafiltration flow was 65 ml/kg/h. A commercial replacement solution (Qingshan Likang, Pharmaceutical Co., Ltd., Chengdu, China) was chosen, and 70–100% of this was pre-diluted. The patients were administered HVHF for 3 consecutive days, during which time the filters were replaced every 24 hours, regardless of whether clotting had occurred. In patients who continued to need RRT beyond the intervention period, intermittent HVHF (12–16 h/d) could be prolonged until the therapeutic purposes were achieved. Regional citrate anticoagulation and regional citrate plus a low dose of low-molecular-weight heparin (LMWH) anticoagulation was preferred for use in the extracorporeal circuit; systemic anticoagulation with only LMWH was also performed occasionally**.**

### Endpoints

The primary endpoints were incidence of sepsis and 90-day mortality. Secondary endpoints were 28-day and 60-day mortality, incidence of septic shock, duration of mechanical ventilation and vasopressor treatment, and length of stay in the intensive care unit (ICU). Adverse events were also recorded. Sepsis was defined according to the 2012 guidelines for the treatment of burn infection [24]. Septic shock was defined as sepsis-induced hypotension persisting with a systolic blood pressure (SBP) < 90 mmHg or a mean arterial pressure (MAP) < 70 mmHg or an SBP decrease > 40 mmHg despite adequate fluid resuscitation [25]. Patients discharged from hospital before the end of the study were followed for 3 months.

The measures of both groups outlined below were recorded at days 1, 3, 5, 7, 14, 21 and 28 post-burn.

1. *Laboratory data*: White blood cell (WBC) count, platelet (PLT) count, potassium (K^+^), sodium (Na^+^), blood glucose (Glu), total bilirubin (TBIL), blood urea nitrogen (BUN), serum creatinine (Cr) of venous blood, and the ratio of arterial oxygen partial pressure to the fraction of inspiration O_2_ (PaO_2_/FiO_2_) of arterial blood were recorded.
2. *Severity of illness*: We assessed severity of illness based on APACHE II and SOFA scores recorded on the day of observation.
3. *Plasma cytokine concentrations and the level of procalcitonin (PCT)*: Venous blood was collected in evacuated tubes containing citric acid, which were centrifuged immediately. The separated plasma was subpackaged and stored at − 80 °C. The plasma cytokine levels (tumour necrosis factor [TNF]-α, interleukin [IL]-1β, IL-6, IL-8, IL-10) were measured using enzyme-linked immunosorbent assay kits according to the manufacturer’s instructions (R&D Systems, Minneapolis, MN, USA). PCT was detected by enzyme-linked fluorescence assay (MINI VIDAS; bioMérieux, Marcy-l’Étoile, France).
4. *Flow cytometry*: Ten healthy adults, 20 patients from the HVHF group and 20 patients from the control group admitted after 2016 were randomly selected and subjected to the following flow cytometric analysis at days 1, 3, 7, 14, 21 and 28 post-burn: expression of human leukocyte antigen (HLA)-DR on CD14^+^ monocytes (eBioscience, San Diego, CA, USA); the proportion of CD4^+^CD25^+^Foxp3^+^ regulatory T cells (Tregs) (eBioscience); and the counts of CD3^+^, CD4^+^ and CD8^+^ T lymphocytes (SemiBioTech, Shanghai, China). Peripheral blood mononuclear cells were obtained by gradient centrifugation using human peripheral blood lymphocyte separating medium (Sangon, Shanghai, China). Cell fluorescence staining was performed according to the protocol included with the kits (eBioscience). After processing, the samples were immediately detected using a flow cytometer (Applied Biosystems Attune; Life Technologies, Carlsbad, CA, USA) and were not fixed and preserved. CD3^+^, CD4^+^ and CD8^+^ T lymphocytes were detected using the CytoCounter device (SemiBio Tech).

### Statistical analysis

According to a previous study, the 90-day mortality of patients with severe burns treated by HVHF in the early stage of burns was 28.6% [26]. However, the 90-day mortality of patients with the same TBSA who received conventional therapy in our institute was 60.0% from 2011 to 2014. The power of the present study with regard to the proportion of 90-day mortality is calculated on the basis of a two-sided chi-square test. With a sample size of 38 subjects treated with HVHF therapy and 38 subjects treated with conventional therapy, the trial had more than 80% power to detect a difference (α = 0.05).

Data of all patients following randomisation were analysed according to the intention-to-treat (ITT) principle. A per-protocol (PP) analysis was also performed to further verify the effects of HVHF treatment. The PP analysis did not include patients discharged against medical advice. Continuous variables are summarised as mean and SD or median and interquartile range (IQR), and categorical variables are summarised as frequency (percent) in each group. The Mann-Whitney *U* test and chi-square test (or Fisher’s exact test) were used to examine differences in the baseline characteristics and outcomes between two groups. Kaplan-Meier survival curves were constructed to compare 90-day survival between the HVHF and control groups via the log-rank test. Repeated measures data were analysed using a linear mixed-effects model. The results were considered significant with *p* values less than 0.05. All statistical analyses were performed using SAS software (version 9.4; SAS Institute, Cary, NC, USA).

## Results

**Fig. 1:**
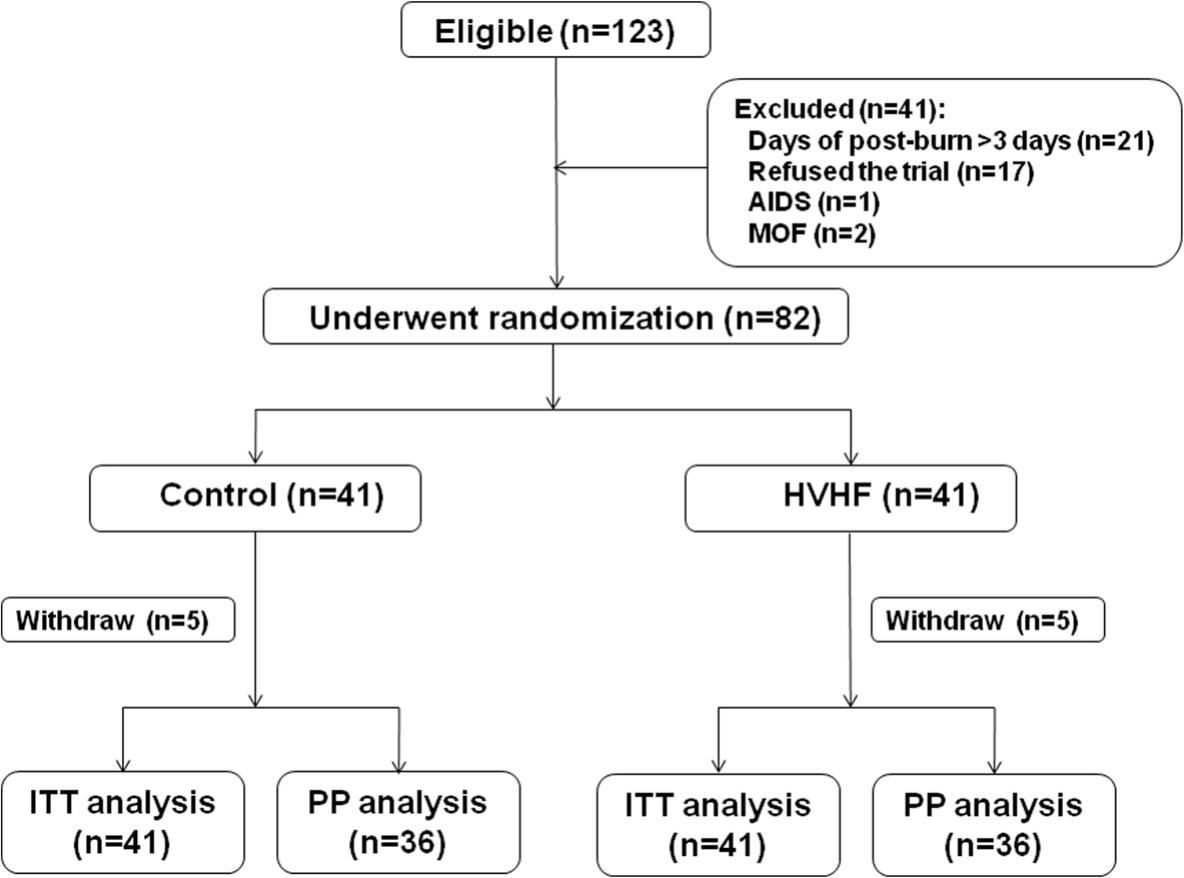
Patient flow diagram. *HVHF* High-volume haemofiltration, *MOF* Multiple organ failure, *AIDS* Acquired immunodeficiency syndrome, *ITT* Intention to treat, *PP* Per protocol

Between March 2014 and October 2017, 123 patients with severe burns were eligible on admission. Forty-one patients were excluded for the following reasons: 21 patients were admitted to our burn centre more than 3 days after the burn, 17 patients refused the trial, 1 patient was diagnosed with acquired immunodeficiency syndrome, and 2 patients developed MOF at admission. A total of 82 patients underwent randomisation. Among them, five patients in each group abandoned therapy and were discharged from hospital during the study, one of whom was in the HVHF group and received therapy for only 1 day. Their median length of stay in hospital was 6 (IQR 5.5, 18.5) days. All the patients were included in the ITT analysis; 10 patients withdrew from the study because they abandoned therapy and were not included in the PP analysis. Figure 1 depicts the flow diagram for the study.

For all randomised patients, the median age was 41.0 ± 11.4 years, and the median TBSA and full-thickness TBSA of the burn were 76.5% (IQR 56.8%, 85.0%) and 35.0% (IQR 20.8%, 54.0%), respectively. The earliest and median HVHF intervention times were 5 hours and 23.0 (IQR 15.5, 34.0) hours after the burn, respectively. HVHF was initiated within 2 hours after randomisation and was performed continuously except for downtime due to changing filters and surgery. The HVHF treatment was usually completed before the first operation. A minority of the patients continued receiving HVHF therapy after the first operation to complete the 3-days treatment. After 3 days of therapy, five patients experienced acute renal failure (ARF) and received RRT (35 ml/kg/h) in the control group for 5 to 16 days. Four patients experienced ARF in the HVHF group; intermittent HVHF was prolonged for 8 to 22 days. Blood purification in the two groups was terminated when renal function was restored or the patient died. ARF was defined as KDIGO (Kidney Disease: Improving Global Outcomes) stage 3 (three or more times baseline or increase in sCr to ≥ 4.0 mg/dl or urinary output < 0.3 ml/kg/h for ≥12 hours). Transitory active bleeding of incisions was found in four patients in the HVHF group. No severe adverse events associated with HVHF occurred. The patients in the two groups had similar baseline characteristics after randomisation, as shown in Table 1. Additionally, the baseline characteristics of patients in PP analysis are presented in Additional file 1: Table S1.

**Table 1:** Baseline characteristics of patients in high-volume haemofiltration and control groups

	Control (*n* = 41)	HVHF (*n* = 41)	*p* Value
Age (years)	42.3 ± 12.0	39.6 ± 10.6	0.29
Gender (% male)	31 (75.6)	34 (83.0)	0.29
BMI (kg/m^2^)	23.7 (20.7, 25.4)	22.0 (20.4, 25.7)	0.46
TBSA (%)	80.0 (60.0, 82.0)	73.0 (55.0, 85.0)	0.79
Full thickness area of burn, TBSA (%)	35.0 (21.0, 49.0)	35.0 (20.5, 58.0)	0.70
ABSI	12.0 (11.0, 13.0)	13.0 (11.0, 14.0)	0.81
Aetiology			0.97
Flame, *n* (%)	29 (70.7)	27 (65.9)	
Scald, *n* (%)	4 (9.8)	4 (9.8)	
Electricity, *n* (%)	7 (17.1)	9 (22.0)	
Chemical, *n* (%)	1 (2.4)	1 (2.4)	
Inhalation injury, *n* (%)	24 (58.5)	27 (65.9)	0.82
Hypovolaemic shock, *n* (%)	17 (41.5)	21 (51.2)	0.87
Receiving MV, *n* (%)	9 (22.0)	6 (14.6)	0.28
Time of randomization (hours post-burn)	20.0 (10.5, 44.8)	22.0 (14.5, 31.0)	0.37
Time of HVHF initiation (hours post-burn)		23.0 (15.5, 34.0)	
APACHE II score	11.0 (9.0, 13.0)	10.0 (9.0, 13.0)	0.51
SOFA score	3.0 (2.0, 5.0)	3.0 (1.5, 4.0)	0.69
TBIL (μmol/L)	15.2 (9.5, 26.6)	17.0 (12.4, 23.7)	0.54
BUN (mmol/L)	7.7 (4.6, 9.7)	6.5 (4.8, 8.6)	0.23
Cr (mmol/L)	88.0 (68.0, 132.0)	78.0 (65.0, 106.0)	0.38
Operation frequency in 28 days post-burn	2.0 (2.0, 2.0)	2.0 (1.0, 3.0)	0.55
Time of first excision (days post-burn)	5.0 (4.0, 7.0)	5.0 (3.5, 5.5)	0.54
Area of first excision, TBSA (%)	18.0 (11.0, 35.0)	18.5 (10.0, 33.5)	0.97
Total area of excision in 28 days post-burn, TBSA (%)	29.0 (19.0, 45.0)	34.0 (20.0, 58.0)	0.42

*Abbreviations: BMI* Body mass index, *TBSA* Total burn surface area, *ABSI* Abbreviated Burn Severity Index, *MV* Mechanical ventilation, *HVHF* High-volume haemofiltration, *APACHE II* Acute Physiology and Chronic Health Evaluation II, *SOFA* Sequential Organ Failure Assessment, *TBIL* Serum total bilirubin, *BUN* Blood urea nitrogen, *Cr* Serum creatinine

Data are presented as mean ± SD, medians (with 25th and 75th quantiles) or percentages. All patients were included in summary tables via the intention-to-treat principle

### Endpoints

As shown in Table 2, the patients in the HVHF group had a significantly lower incidence of sepsis (26.8% vs. 51.2%, *p* = 0.04) and septic shock (14.6% vs. 43.9%, *p* = 0.01), as well as a shorter duration of vasopressor treatment (1.0 d vs. 4.0 d, *p* < 0.001), than those in the control group (Table 2), although no significant difference was found in 28-, 60- and 90-day mortality, duration of mechanical ventilation or ICU days. Among the ten patients discharged against medical advice, one patient in the control group was treated in another hospital and survived until the end of the 90-day follow-up period, and the other nine patients died.

**Table 2:** Outcomes of patients in high-volume haemofiltration and control groups

	Burn ≥ 50% TBSA			Burn ≥ 80% TBSA		
	Control (*n* = 41)	HVHF (*n* = 41)	*p* Value	Control (*n* = 21)	HVHF (*n* = 17)	*p* Value
Mortality						
28-day, *n* (%)	13 (31.7)	9 (22.0)	0.46	10 (47.6)	5 (29.4)	0.33
60-day, *n* (%)	17 (41.5)	11 (26.8)	0.24	13 (61.9)	5 (29.4)	0.06
90-day, *n* (%)	19 (46.3)	11 (26.8)	0.11	14 (66.7)	5 (29.4)^a^	0.049
Sepsis, *n* (%)	21 (51.2)	11 (26.8)^a^	0.04	15 (71.4)	6 (37.5)^a^	0.04
Septic shock, *n* (%)	18 (43.9)	6 (14.6)^a^	0.01	13 (61.9)	3 (18.6)^a^	0.01
Duration of MV (days)	8.5 (5.3, 11.8)	7.0 (4.0, 10.5)	0.19	9.5 (2.5, 16.3)	7.0 (2.0, 8.0)	0.09
ICU days	28.5 (20.0, 48.0)	34.5 (15.5, 55.0)	0.84	31.0 (21.5, 47.5)	49.0 (18.0, 67.0)	0.23
Duration of vasopressors (days)	4.0 (2.0, 11.0)	1.0 (1.0, 1.0)^a^	0.001	8.0 (2.0, 12.5)	1.5 (1.0, 2.8)^a^	0.04

*Abbreviations: TBSA* Total burn surface area, *MV* Mechanical ventilation, *ICU* Intensive care unit, *HVHF* High-volume haemofiltration

Data are presented as medians (25th and 75th quantiles) or percentages. All patients were included in outcome comparisons via the intention-to-treat principle

^a^ *p* < 0.05 indicates a significant difference compared with the control group

**Fig. 2:**
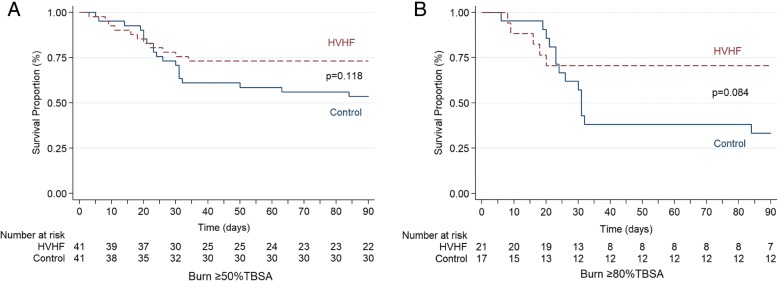
Kaplan-Meier estimate of 90-day survival in the high-volume haemofiltration (HVHF) and control groups at the indicated time points. **a** Patients with burns ≥ 50% total burn surface area (TBSA). **b** Patients with burns ≥ 80% TBSA

However, in the subset of patients with extremely severe burns (burns ≥ 80% TBSA, which were classified as the most serious injuries in our burn centre), the primary endpoints—incidence of sepsis (37.5% vs. 71.4%, *p* = 0.04) and 90-day mortality (29.4% vs. 66.7%, *p* = 0.049)—both showed significant decreases in the HVHF group compared with the control group (Table 2). The same benefits were present in the secondary endpoints: incidence of septic shock (18.6% vs. 61.9%, *p* = 0.01) and duration of vasopressor treatment (1.5 d vs. 8.0 d, *p* = 0.04) (Table 2). The Kaplan-Meier survival curve demonstrated no significant differences in 90-day survival between the two groups on the basis of the log-rank test (Fig. 2).

Additionally, a PP analysis showed a significant decrease in 90-day mortality (16.7% vs. 41.7%, *p* = 0.04) in the patients with burns ≥ 50% TBSA in the HVHF group compared with the control group (Table 3). Similarly, the PP analysis further verified the superiority of HVHF in the patients with burns ≥ 80% TBSA regarding the incidence of sepsis (37.5% vs. 76.5%, *p* = 0.04) and 90-day mortality (25.0% vs. 64.7%, *p* = 0.04) compared with conventional treatment only (Table 3).

**Table 3:** Outcomes of patients in high-volume haemofiltration and control groups in per-protocol analysis

	Burn ≥ 50% TBSA			Burn ≥ 80% TBSA		
	Control (*n* = 36)	HVHF (*n* = 36)	*p* Value	Control (*n* = 17)	HVHF (*n* = 16)	*p* Value
Mortality						
28-day, *n* (%)	9 (25.0)	5 (13.9)	0.37	7 (41.2)	4 (25.0)	0.46
60-day, *n* (%)	13 (36.1)	6 (16.7)	0.11	10 (58.8)	4 (25.0)	0.08
90-day, *n* (%)	15 (41.7)	6 (16.7)^a^	0.04	11 (64.7)	4 (25.0)^a^	0.04
Sepsis, *n* (%)	19 (52.8)	10 (27.8)	0.05	13 (76.5)	6 (37.5)^a^	0.04
Septic shock, *n* (%)	16 (44.4)	5 (13.9)^a^	0.01	11 (64.7)	3 (18.8)^a^	0.01
Duration of MV (days)	9.5 (7.0, 11.8)	7.0 (5.0, 10.0)	0.06	10.0 (7.0, 18.0)	7.0 (2.0, 8.0)^a^	0.03
ICU days	30.0 (20.5, 48.8)	34.0 (15.5, 53.0)	0.70	31.0 (24.5, 54.0)	51.5 (22.3, 78.3)	0.26
Duration of vasopressors (days)	4.0 (2.0, 11.3)	1.0 (1.0, 2.0)^a^	0.005	2.0 (4.0, 11.3)	1.0 (1.0, 2.0)^a^	0.005

*Abbreviations: TBSA* Total burn surface area, *MV* Mechanical ventilation, *ICU* Intensive care unit, *HVHF* High-volume haemofiltration

Data are presented as mean ± SD, median (25th and 75th quantiles) or percentages. Patients with complete data were included in outcomes comparison via the per-protocol analysis

^a^ *p* < 0.05 indicates a significant difference compared with the control group

### Laboratory data

Over the 28-day observation period, compared with the control group, the data for PaO_2_/FiO_2_ were significantly higher (*p* < 0.001) in the patients in the HVHF group, although no significant difference was found in the WBC and PLT counts or in the concentrations of TBIL, K^+^, Na^+^ and GLU (Additional file 2: Figure S1).

### APACHE II score and SOFA score

**Fig. 3:**
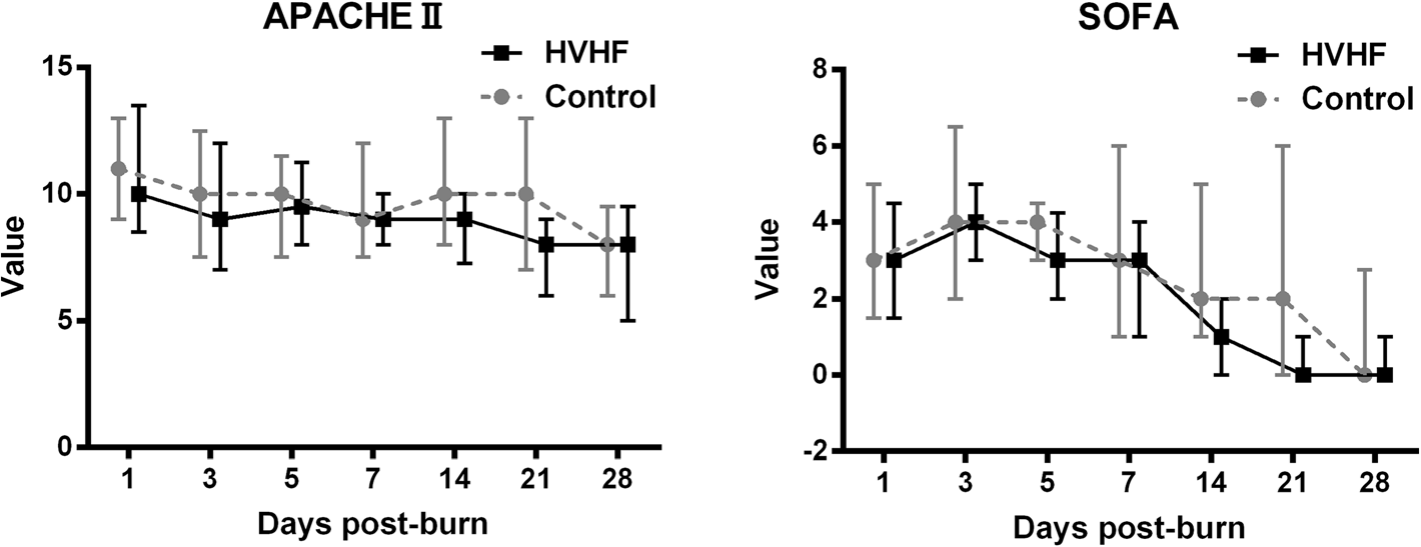
Acute Physiology and Chronic Health Evaluation II (APACHE II) score and Sequential Organ Failure Assessment (SOFA) score of the patients in the high-volume haemofiltration (HVHF) and control groups at the indicated time points

As shown in Fig. 3, the APACHE II score and SOFA score of the patients in the HVHF group showed a continuous decreasing trend and were significantly lower than those of the patients in the control group over time (*p* = 0.01 and *p* = 0.02, respectively).

### Cytokine profile and PCT level

**Fig. 4:**
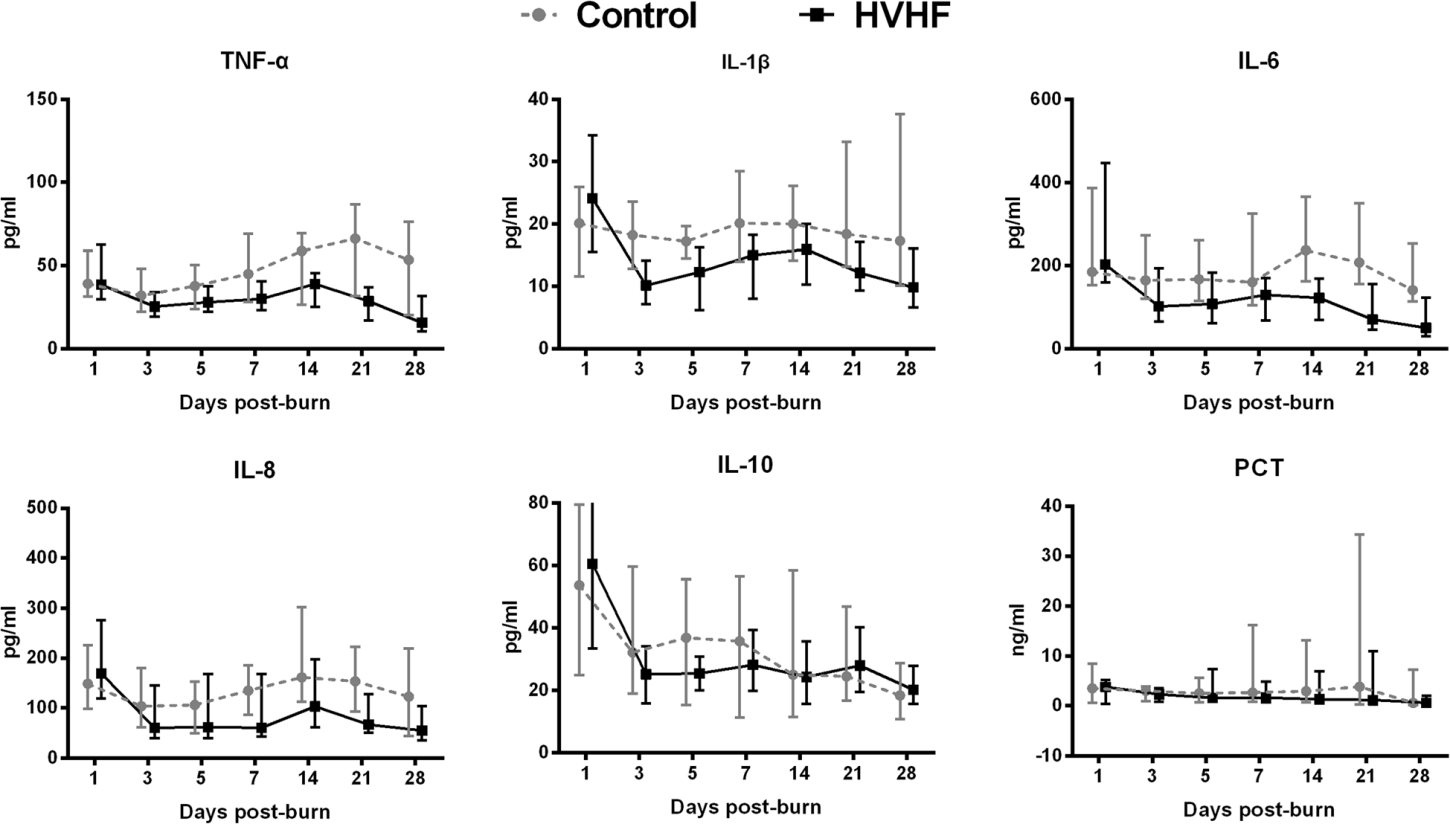
Inflammatory cytokine and PCT levels in the blood of patients in the HVHF and control groups at the indicated time points. *IL* Interleukin, *TNF* Tumour necrosis factor, *PCT* Procalcitonin, *HVHF* High-volume haemofiltration

In the HVHF group, significant lower levels were found in plasma cytokines, including TNF-α (*p* = 0.003), IL-1β (*p* = 0.01), IL-6 (*p* = 0.02), IL-8 (*p* = 0.04) and PCT (*p* = 0.005), than in the control group (Fig. 4). No significant difference was observed in the plasma cytokine IL-10 by linear mixed-effect model analysis over time (Fig. 4).

### Flow cytometry

**Fig. 5:**
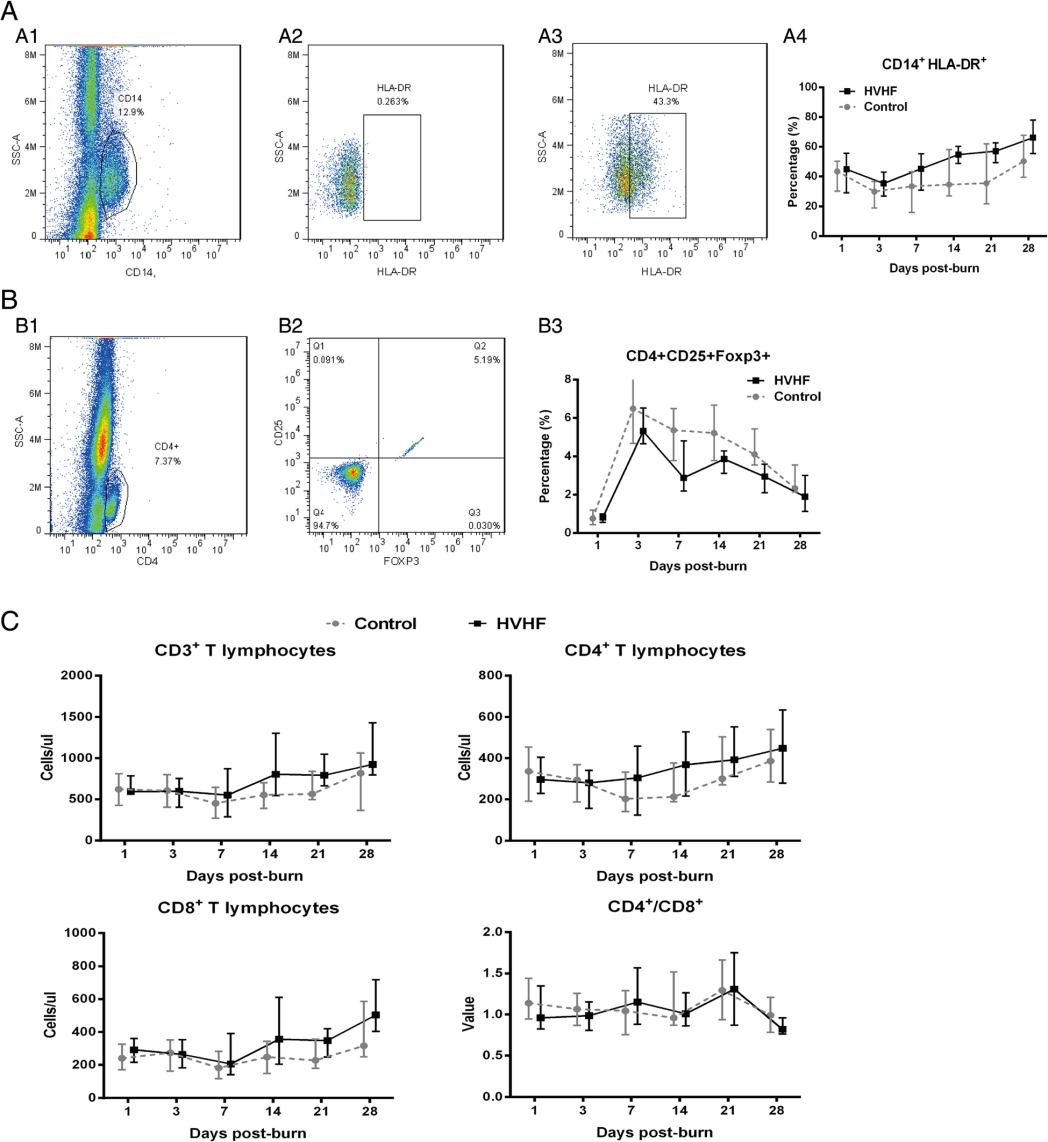
Flow cytometric analysis in the high-volume haemofiltration (HVHF) and control groups at the indicated time points. **a** Expression of HLA-DR on CD14^+^ monocytes. (A1) Gating strategy for CD14^+^ cells. (A2) Isotype controls for HLA-DR. (A3) Detected expression of HLA-DR on CD14^+^ monocytes. **b** Proportion of CD4^+^CD25^+^Foxp3^+^ regulatory T lymphocytes. (B1) Gating strategy for CD4^+^ cells. (B2) Detected proportion of CD25^+^Foxp3^+^ in CD4^+^ cells. **c** Counts of CD3^+^, CD4^+^ and CD8^+^ T lymphocytes

All patients were characterised by reduced expression of HLA-DR on peripheral blood CD14^+^ monocytes. Compared with healthy adults, patients in both groups had HLA-DR expression on the CD14^+^ monocytes that decreased to the lowest level at day 3 post-burn (*p* < 0.0001). Then the level of HLA-DR expression gradually recovered from day 7 to day 28 post-burn. It is worth noting that HLA-DR expression recovered to a greater extent in the HVHF patients than in the control patients (*p* = 0.03) (Fig. 5A4). The proportion of CD25^+^Foxp3^+^ in CD4^+^ cells increased to the highest level at day 3 post-burn (*p* < 0.0001) and then gradually decreased from days 7 to 28 post-burn in the two groups; compared with the control group, the patients in the HVHF group had a significantly lower proportion (*p* = 0.04) (Fig. 5B3). From day 1 after burn, the counts of CD3^+^, CD4^+^ and CD8^+^ T lymphocytes in both the HVHF and control groups were significantly lower than in healthy adult**s** (*p* < 0.05), and they showed no difference between the two groups over time (*p* > 0.05) (Fig. 5C).

## Discussion

Sepsis is defined as a life-threatening organ dysfunction caused by a dysregulated host response to infection [1]. Owing to excessive inflammation, early sepsis often results in a high level of inflammatory mediators in the blood, which can lead to organ injury and dysfunction [27]. In addition, the prolonged release of inflammatory mediators can cause immunocyte impairment and further immunosuppression [28], and it can even evolve into immune paralysis and increase the chance of secondary infection, ultimately causing death [29]. Therefore, early preventive measures to control excessive inflammatory mediators, alleviate inflammation and maintain immune homeostasis can reduce the incidence of sepsis and organ dysfunction, thus improving patient outcomes.

Extracorporeal blood purification can remove a broad spectrum of inflammatory mediators and bacterial toxins in a non-selective way through filtration and adsorption and has been proposed to improve the prognosis of patients with sepsis [9]. Several theories have been proposed to explain the potential benefits of blood purification in sepsis. First, the “peak concentration hypothesis” [30] suggests that blood purification therapy can lower the overall level of water-soluble mediators at the pro-inflammatory stage and, owing to the peak-shaving effects, can decrease the incidence of MOF syndrome and reduce mortality. Second, the “threshold immunomodulation hypothesis” [31] explains that some cytokines equilibrate between the blood and tissue compartments. Therefore, blood purification clears mediators in the blood directly, and the mediators in the interstitium and tissues will then be indirectly reduced owing to the concentration gradient. When a certain threshold value is reached, inflammatory cascade reactions are blocked and organ injuries are attenuated. Third, the “mediator delivery hypothesis” [32] proposes that HVHF can reinforce the lymphatic flow in mediator-rich tissues and displace inflammatory mediators to the blood compartment, from whence they are gradually removed. The fourth hypothesis considers the effect of regulating and maintaining immunological homeostasis [15]. A previous study demonstrated that HVHF reverses sepsis-related immunoparalysis in a porcine model of pancreatitis and increases the expression of major histocompatibility complex II and CD14 in monocytes, thereby alleviating oxidative stress and improving the phagocytic ability of polymorphonuclear leukocytes, even reducing bacterial translocation and endotoxaemia [33]. Ronco [7] also proposed that HVHF and coupled plasma filtration adsorption regulate the immunological functions of sepsis.

However, treating sepsis by blood purification remains controversial. Some studies have found that blood purification, including the use of polymyxin B haemoperfusion [34], cannot lower the level of blood inflammatory mediators and does not improve the survival rate of patients with sepsis [11, 35–37]. The Surviving Sepsis Campaign guidelines panel has noted that there is inadequate research evidence in favour of or against the use of blood purification to treat patients with sepsis, and the clinical benefit of blood purification requires further clarification [13]. However, some beneficial effects have been found in patients with severe burns. Chung et al. revealed that HVHF reversed shock and improved organ function in burn patients with septic shock and AKI [37]. Early and aggressive CVVH decreased mortality in patients with burns and critically ill patients [16, 26]. In addition, therapeutic plasma exchange may be effective as a salvage intervention for refractory burn shock [38]. These inconsistent results may be explained by the different pathophysiological features between patients with severe burns and general ICU patients. In general, the evidence suggests that blood purification is beneficial for patients with severe burns.

In this study, we observed that HVHF in the early stage of severe burn reduced the incidence of sepsis and septic shock in patients with burns ≥ 50% TBSA. Notably, it additionally improved the survival of patients with burns ≥ 80% TBSA. In our centre, we assess the severity of burns based largely on the burn depth, burn size and burn-related complications. Because it remains severely challenging to save patients with burns ≥ 80% TBSA, these burns are classified as extremely severe. No differences were found in the duration of mechanical ventilation and ICU days between the two groups. This may have occurred because more critically ill patients survived, prolonging the two variables in the HVHF group. For example, the two surviving patients with 95% TBSA (deep partial thickness burn 32% TBSA, full-thickness burn 65%TBSA) and 95%TBSA (all full-thickness burns) extended their ICU stays to 75 and 78 days, respectively. A PP analysis was performed and also showed that HVHF treatment was effective in reducing the incidence of sepsis and 90-day mortality of the patients with severe burns. No severe adverse events were observed during HVHF treatment. Early application of HVHF may be a key treatment technology for treating severe burns.

Patients with extensive burns presented manifestations of prolonged and intense inflammatory responses [3, 39]. We found that some inflammatory cytokines in the blood increased immediately after the burns, and this may be related to early stress reactions. The highest levels of most inflammatory cytokines appeared from days 7 to 21 post-burn and may have been closely associated with the dissolving of eschar, wound infection or sepsis occurring in the patients. The early excessive inflammatory response is mainly caused by the burn wound and has the primary role in the initiation of organ injury. Thus, early blood purification treatment combined with effective wound management, including debridement to remove necrotic tissue, wound closure by skin grafting, and skin substitute covering, might stop the cascading inflammatory response, and this may be beneficial in improving patient prognosis. In the HVHF group, the lower levels of most inflammatory cytokines in the circulation contributed to alleviating the inflammatory reactions and may have subsequently improved PaO_2_/FiO_2_ and organ function (represented by SOFA score).

The decreases in inflammatory cytokine levels after HVHF found in this study may have occurred in two ways: The first is the direct effect of haemofiltration and adsorption, and the second is the massive removal of harmful substances, such as stress hormones, oxygen free radicals, inflammatory mediators and metabolites, which may indirectly reduce inflammatory cytokine production. In this study, the cytokine levels were not reduced in a small number of patients after HVHF treatment, possibly owing to the severe infection and immune response status of these patients, who exhibited uncontrolled cytokine production from blood infections or other infectious foci beyond the clearance capacity of haemofiltration.

In addition to early changes in inflammatory mediators, changes in immune status may also be closely related to sepsis. Many studies have shown that the immunosuppression of sepsis and severe trauma is associated with lymphocyte apoptosis or immune cell dysfunction [40, 41]. Immune imbalance and a lack or delay of the anti-inflammatory response may be an important cause of burn sepsis [26, 40]. In addition to clearing inflammatory mediators, blood purification plays a role in the regulation of immune status [42]. HLA-DR expression on CD14^+^ monocytes reflects the antigen-presenting ability of monocytes and is considered a reliable marker for evaluating the immune function of critically ill patients [43]. Furthermore, the activation and expansion of CD4^+^CD25^+^ T cells contributes to the development of immunosuppression and sepsis after severe burns [44]. In this study, we found that HVHF treatment promoted the recovery of HLA-DR expression on CD14^+^ monocytes and the counts of CD3, CD4 and CD8 T lymphocytes in patients with burns. Moreover, early HVHF treatment inhibits the excessive increase of Tregs. These findings indicate that secondary immune deficiency occurs in patients with severe burns and that early HVHF treatment may facilitate the recovery of immune function owing to the clearance of cytokines, consequently reducing the incidence of sepsis and sepsis-related mortality. However, further studies are needed to confirm these results.

Our previous studies showed that the use of CVVH in treating burn sepsis did not improve patient survival [12, 13]. Over the past 5 years, our ideas related to the application of blood purification have undergone some changes: (1) from CVVH with a standard renal dose of 35 ml/kg/h to HVHF at a dose of 65 ml/kg/h, (2) from renal replacement therapy or treating sepsis to the prevention of sepsis and organ dysfunction at an early stage after burns, (3) from the use of systemic heparin or LMWH anticoagulation treatments to regional citrate anticoagulation, and (4) from intermittent therapy to continuous treatment. In the present study, we observed that early continuous HVHF benefited patients with severe burns owing to early clearance of inflammatory mediators, recovery of immune status and protection of the internal environment and organ function.

However, this study had some limitations. Firstly, these preliminary findings are from our single burn centre. A multicentre study is needed to verify these results. Secondly, although all the enrolled patients received interventions within 3 days post-burn, some of the patients might have received different treatments before being admitted to our centre, which may have had some influence on the results. Thirdly, the definition of sepsis used in our study was based on the 2012 guidelines for the treatment of burn infection, which may have had some subtle differences from the criteria used in general ICU patents. In addition, in the present study, numerous aspects, including the optimal mode, dose, timing, duration and frequency of HVHF in the early stage after severe burn, remain to be studied.

## Conclusions

Early application of HVHF benefits patients with severe burns, especially those with a greater burn area (≥ 80% TBSA), decreases the incidence of sepsis and mortality. This effect may be attributed to early clearance of inflammatory mediators and the recovery of the patient’s immune status. Thus, in patients with severe burns, HVHF treatment is effective and safe and should be used as an active intervention rather than as a passive organ support treatment for organ failure, especially for septic shock with MODS.

## Additional files

Additional file 1: **Table S1.** Baseline characteristics of the patients in the HVHF and control groups for PP analysis. (DOCX 16 kb)

Additional file 2: **Figure S1.** Laboratory data of the patients in the HVHF and control groups at the indicated time points. *WBC* White blood cell count, *GLU* Glucose, *TBIL* Serum total bilirubin, *Cr* Serum creatinine, *BUN* Blood urea nitrogen, *PaO*_*2*_*/FiO*_*2*_ Ratio of arterial oxygen partial pressure to fraction of inspired oxygen, *PLT* Platelet count, *HVHF* High-volume haemofiltration. (TIF 115 kb)

## Abbreviations

ABSI: Abbreviated Burn Severity Index
AKI: Acute kidney injury
APACHE II: Acute Physiology and Chronic Health Evaluation II
ARF: Acute renal failure
BMI: Body mass index
BUN: Blood urea nitrogen
CVVH: Continuous venovenous haemofiltration
Glu: Blood glucose
HLA-DR: Human leukocyte antigen DR
HVHF: High-volume haemofiltration
ICU: Intensive care unit
IL: Interleukin
IQR: Interquartile range
LMWH: Low-molecular-weight heparin
MODS: Multiple organ dysfunction syndrome
PaO_2_/FiO_2_: Ratio of arterial oxygen partial pressure to the fraction of inspiration oxygen
PCT: Procalcitonin
PLT: Platelet
RRT: Renal replacement therapy
sCr: Serum creatinine
SOFA: Sequential Organ Failure Assessment
TBIL: Total bilirubin
TBSA: Total burn surface area
TNF: Tumour necrosis factor
Treg: Regulatory T cells
WBC: White blood cell

## References

[1] Rhodes A, Evans LE, Alhazzani W (2017). Surviving Sepsis Campaign: international guidelines for management of sepsis and septic shock: 2016. Intensive Care Med.

[2] Finnerty CC, Herndon DN, Przkora R (2006). Cytokine expression profile over time in severely burned pediatric patients. Shock.

[3] Finnerty CC, Jeschke MG, Herndon DN (2008). Temporal cytokine profiles in severely burned patients: a comparison of adults and children. Mol Med.

[4] Hotchkiss RS, Monneret G, Payen D (2013). Immunosuppression in sepsis: a novel understanding of the disorder and a new therapeutic approach. Lancet Infect Dis.

[5] Limaye AP, Kirby KA, Rubenfeld GD (2008). Cytomegalovirus reactivation in critically ill immunocompetent patients. JAMA.

[6] Guzman N, Podoll AS, Bell CS (2013). Myoglobin removal using high-volume high-flux hemofiltration in patients with oliguric acute kidney injury. Blood Purif.

[7] Ronco C (2017). Continuous renal replacement therapy: forty-year anniversary. Int J Artif Organs.

[8] Bouman CS, Oudemans-van SHM, Schultz MJ (2007). Hemofiltration in sepsis and systemic inflammatory response syndrome: the role of dosing and timing. J Crit Care.

[9] Rimmele T, Kellum JA (2011). Clinical review: blood purification for sepsis. Crit Care.

[10] Shum HP, Yan WW, Chan TM (2016). Extracorporeal blood purification for sepsis. Hong Kong Med J.

[11] Borthwick EM, Hill CJ, Rabindranath KS (2017). High-volume haemofiltration for sepsis in adults. Cochrane Database Syst Rev.

[12] Hu G, Peng Y, Wang F, Zhu M, Gong Y (2014). Effects of blood purification in the treatment of patients with the burn sepsis [in Chinese]. Zhonghua Shao Shang Za Zhi.

[13] Peng Y, Yuan Z, Li H (2005). Removal of inflammatory cytokines and endotoxin by veno-venous continuous renal replacement therapy for burned patients with sepsis. Burns.

[14] Balestra GM, Legrand M, Ince C (2009). Microcirculation and mitochondria in sepsis: getting out of breath. Curr Opin Anaesthesiol.

[15] Linden K, Stewart IJ, Kreyer SF (2014). Extracorporeal blood purification in burns: a review. Burns.

[16] Chung KK, Lundy JB, Matson JR (2009). Continuous venovenous hemofiltration in severely burned patients with acute kidney injury: a cohort study. Crit Care.

[17] Honore PM, Joannes-Boyau O, Boer W (2009). High-volume hemofiltration in sepsis and SIRS: current concepts and future prospects. Blood Purif.

[18] Vincent JL, de Mendonca A, Cantraine F (1998). Use of the SOFA score to assess the incidence of organ dysfunction/failure in intensive care units: results of a multicenter, prospective study. Working group on “sepsis-related problems” of the European Society of Intensive Care Medicine. Crit Care Med.

[19] Knaus WA, Draper EA, Wagner DP (1985). APACHE II: a severity of disease classification system. Crit Care Med.

[20] Li H, Wang S, Tan J (2017). Epidemiology of pediatric burns in Southwest China from 2011 to 2015. Burns.

[21] Li H, Zhou J, Peng Y (2017). The progress of Chinese burn medicine from the Third Military Medical University—in memory of its pioneer, Professor Li Ao. Burns Trauma.

[22] Luo G, Peng Y, Yuan Z (2009). Fluid resuscitation for major burn patients with the TMMU protocol. Burns.

[23] Luo G, Fan H, Sun W (2011). Blood loss during extensive escharectomy and auto-microskin grafting in adult male major burn patients. Burns.

[24] Yizhi P, Jing C, Zhiqiang Y (2013). Diagnostic criteria and treatment protocol for post-burn sepsis. Crit Care.

[25] Dellinger RP, Levy MM, Rhodes A (2013). Surviving Sepsis Campaign: international guidelines for management of severe sepsis and septic shock, 2012. Intensive Care Med.

[26] F J, H R, D G (2016). Continuous blood purification therapy in severe burn patients [in Chinese]. Shen Zang Bing Yu Tou Xi Shen Yi Zhi Za Zhi.

[27] Van der Poll T, van de Veerdonk FL, Scicluna BP (2017). The immunopathology of sepsis and potential therapeutic targets. Nat Rev Immunol.

[28] Hotchkiss RS, Coopersmith CM, McDunn JE (2009). The sepsis seesaw: tilting toward immunosuppression. Nat Med.

[29] Luyt CE, Combes A, Deback C (2007). Herpes simplex virus lung infection in patients undergoing prolonged mechanical ventilation. Am J Respir Crit Care Med.

[30] Ronco C, Tetta C, Mariano F (2003). Interpreting the mechanisms of continuous renal replacement therapy in sepsis: the peak concentration hypothesis. Artif Organs.

[31] Honoré PM, Matson JR (2004). Extracorporeal removal for sepsis: acting at the tissue level—the beginning of a new era for this treatment modality in septic shock. Crit Care Med.

[32] Di Carlo JV, Alexander SR (2005). Hemofiltration for cytokine-driven illnesses: the mediator delivery hypothesis. Int J Artif Organs.

[33] Yekebas EF, Eisenberger CF, Ohnesorge H (2001). Attenuation of sepsis-related immunoparalysis by continuous veno-venous hemofiltration in experimental porcine pancreatitis. Crit Care Med.

[34] Payen DM, Guilhot J, Launey Y (2015). Early use of polymyxin B hemoperfusion in patients with septic shock due to peritonitis: a multicenter randomized control trial. Intensive Care Med.

[35] Joannes-Boyau O, Honore PM, Perez P (2013). High-volume versus standard-volume haemofiltration for septic shock patients with acute kidney injury (IVOIRE study): a multicentre randomized controlled trial. Intensive Care Med.

[36] Payen D, Mateo J, Cavaillon JM (2009). Impact of continuous venovenous hemofiltration on organ failure during the early phase of severe sepsis: a randomized controlled trial. Crit Care Med.

[37] Chung KK, Coates EC, Smith DJ (2017). High-volume hemofiltration in adult burn patients with septic shock and acute kidney injury: a multicenter randomized controlled trial. Crit Care.

[38] Neff LP, Allman JM, Holmes JH (2010). The use of therapeutic plasma exchange (TPE) in the setting of refractory burn shock. Burns.

[39] Jeschke MG, Mlcak RP, Finnerty CC (2007). Burn size determines the inflammatory and hypermetabolic response. Crit Care.

[40] Nitzschke SL, Aden JK, Serio-Melvin ML (2014). Wound healing trajectories in burn patients and their impact on mortality. J Burn Care Res.

[41] Drewry AM, Samra N, Skrupky LP (2014). Persistent lymphopenia after diagnosis of sepsis predicts mortality. Shock.

[42] Gong D, Zhang P, Ji D (2010). Improvement of immune dysfunction in patients with severe acute pancreatitis by high-volume hemofiltration: a preliminary report. Int J Artif Organs.

[43] Volk HD, Reinke P, Krausch D (1996). Monocyte deactivation—rationale for a new therapeutic strategy in sepsis. Intensive Care Med.

[44] Huang LF, Yao YM, Dong N (2010). Association between regulatory T cell activity and sepsis and outcome of severely burned patients: a prospective, observational study. Crit Care.

