# Supplementary material for: Early application of continuous high-volume haemofiltration can reduce sepsis and improve the prognosis of patients with severe burns
Source: Crit Care. 2018 Jul 6;22:173. doi: 10.1186/s13054-018-2095-9 (PMC6035411; doi:10.1186/s13054-018-2095-9)
Supplement: Supplementary file 1 — Table S1. Baseline characteristics of the patients in the HVHF and control groups for PP analysis. (DOCX 16 kb) [file 13054_2018_2095_MOESM1_ESM.docx]

| **Additional file 1 Table S1. Baseline characteristics of the patients in the HVHF and control groups for PP analysis** | | | |
| --- | --- | --- | --- |
|  | Control (n = 36) | HVHF (n = 36) | P value |
| Age (years) | 42.4±11.9 | 40.2±10.7 | 0.41 |
| Gender (% male) | 27 (75.0) | 30 (83.3) | 0.56 |
| BMI (kg/m^2^) | 23.2 (20.7, 24.9) | 22.1 (20.5, 25.7) | 0.88 |
| TBSA (%) | 76.5 (45.0, 82.0) | 72.5 (54.3, 88.8) | 0.90 |
| Full thickness area of burn (%) | 29.0 (20.0, 46.3) | 29.5 (22.1, 47.5) | 0.57 |
| ABSI | 12.0 (11.0, 13.0) | 12.0 (10.3, 14) | 0.87 |
| Etiology |  |  | 0.72 |
| Flame, n (%) | 23 (63.9) | 27 (75.0) |  |
| Scald, n (%) | 4 (11.1) | 4 (11.1) |  |
| Electricity, n (%) | 8 (22.2) | 4 (11.1) |  |
| Chemical, n (%) | 1 (2.8) | 1 (2.8) |  |
| Inhalation injury, n (%) | 21 (58.3) | 23 (63.9) | 0.81 |
| Hypovolemic shock, n (%) | 15 (41.7) | 17 (41.2) | 0.81 |
| Receiving MV, n (%) | 6 (16.7) | 4 (11.1) | 0.74 |
| Time of randomization (hours post-burn) | 20.0 (10.0, 24.0) | 21.5 (13.3, 31.0) | 0.37 |
| Time of HVHF initiation (hours post-burn) |  | 23.5 (15.0, 34.5) |  |
| APACHE II score | 11 (9,13) | 10 (8, 12.5) | 0.51 |
| SOFA score | 3 (1,5.3) | 3.5 (2, 9) | 0.10 |
| TBIL (µmol/L) | 15.7 (12.4, 22.5) | 15.2 (10.6, 27.3) | 0.72 |
| BUN (mmol/L) | 7.1 (4.4,10) | 6.6 (4.8, 10.2) | 0.74 |
| Cr (mmol/L) | 89 (67.5, 111.8) | 78 (65, 106) | 0.64 |
| Operation frequency in 28 days post-burn | 2.0 (1.5, 2.5) | 2.0 (1.0, 3.0) | 0.54 |
| Time of first excision (days post-burn) | 5.0 (3.5, 7.0) | 4.5 (3.0, 5.3) | 0.51 |
| Area of first excision, TBSA (%) | 16.0 (8.5, 34.5) | 18.0 (5.3, 29.5) | 0.56 |
| Total area of excision in 28 days post-burn, TBSA (%) | 28.0 (17.5, 46.5) | 26.0 (14.0, 55.0) | 0.95 |

Data are presented as mean ± SD, median (with 25th and 75th quantiles) or percentages. BMI, Body Mass Index; TBSA, total burn surface area. ABSI, Abbreviated Burn Severity Index; MV mechanical ventilation; HVHF high-volume haemofiltration; APACHEII, Acute Physiology and Chronic Health Evaluation II; SOFA, Sequential Organ Failure Assessment; TBIL, serum total bilirubin; BUN, blood ureanitrogen; Cr, serum creatinine
